# A Review of Deep Learning-Based Methods for Pedestrian Trajectory Prediction

**DOI:** 10.3390/s21227543

**Published:** 2021-11-13

**Authors:** Bogdan Ilie Sighencea, Rareș Ion Stanciu, Cătălin Daniel Căleanu

**Affiliations:** Applied Electronics Department, Faculty of Electronics, Telecommunications, and Information Technologies, Politehnica University Timișoara, 300223 Timișoara, Romania; bogdan.sighencea@student.upt.ro (B.I.S.); rares.stanciu@upt.ro (R.I.S.)

**Keywords:** trajectory prediction, pedestrian behavior, autonomous vehicles, sensor technologies, deep learning

## Abstract

Pedestrian trajectory prediction is one of the main concerns of computer vision problems in the automotive industry, especially in the field of advanced driver assistance systems. The ability to anticipate the next movements of pedestrians on the street is a key task in many areas, e.g., self-driving auto vehicles, mobile robots or advanced surveillance systems, and they still represent a technological challenge. The performance of state-of-the-art pedestrian trajectory prediction methods currently benefits from the advancements in sensors and associated signal processing technologies. The current paper reviews the most recent deep learning-based solutions for the problem of pedestrian trajectory prediction along with employed sensors and afferent processing methodologies, and it performs an overview of the available datasets, performance metrics used in the evaluation process, and practical applications. Finally, the current work exposes the research gaps from the literature and outlines potential new research directions.

## 1. Introduction

Globally, pedestrians represent 23% of the worldwide 1.35 million road traffic deaths every year [1]. Most of these tragic events happen in crowded spaces at pedestrian crossings in poor visibility conditions due to the drivers’ diminished attention. In an eventual impact, pedestrians have virtually no protection. Therefore, reducing (eliminating) these impacts is an important safety issue. Helping the driver in such conditions includes predicting the pedestrian’s trajectory and/or behavior and mitigating the driver’s consequential errors (e.g., tiredness, inadvertent cognitive distraction) and includes developing new technologies to decrease the number of accidents (by up to 93.5%, according to [2]). Human error impact reduction can improve the safety of the driver of the vehicle and other traffic participants such as pedestrians. Over half of these accidents are reported to occur on rural roads (55%), while urban areas are responsible for a lower events percentage (37%) [3]. Half of all accidents involving pedestrians occur at night. According to [4], low-light conditions are believed to be the main cause of these accidents.

According to [5], the autonomous vehicle concept should address the safety concerns for what is called “vulnerable road users”. Because they benefit from little to no protection, pedestrians and cyclists qualify as vulnerable. While pedestrian trajectory prediction (PTP) applications have become increasingly applicable, this area has recently gained importance. For example, the development of social robot platforms and autonomous driving is based on predictions of pedestrian trajectory, which relies on the previous steps of the pedestrians to predict their paths. This prediction takes into account the interactions with other pedestrians of a certain person walking toward a destination. More precisely, pedestrians walk with their companions by following the footmarks left by their surrounding flows. In addition, they tend to prevent collisions by choosing the best route. Each pedestrian is characterized by a particular pattern of motion, which depends on their gaits, acceleration, and velocities. It is necessary to develop a system that can use the first observations related to a person with the aim of understanding and learning some person-specific movement properties. If there are some pedestrians present at the scene, their coordinates need to be observed for a certain period of time (in seconds). The next coordinate of each person must be predicted from a defined period and time estimation. The pedestrian coordinates (in meters) describe the exact location of a person, having, as a landmark, a fixed point that is unique for each scene and that is arbitrarily chosen.

Pedestrian trajectory prediction is a complex task because humans may change directions suddenly depending on objects, vehicles, human interaction, etc. Human behavior may change due to events occurring (suddenly) within the scene [6]. For example, if the bus arrives, people who intended to take that bus tend to move faster (even suddenly starting to run if previously walking) to catch it. A more complex scene increases the difficulty of PTP. In the perfect scenario, the walking destination of the achievement of the pedestrian leads to the trajectory pattern’s achievement. However, in the real world, the destination of the pedestrian is not always known. The trajectory pattern should be inferred based on past trajectory sequence. The automotive component responsible for increasing car and road safety and reducing road fatalities is the advanced driver assist system (ADAS). Without the help of deep learning methods, the ADAS system alone may not always be able to find the destination of the pedestrian.

Different approaches were used to try to develop systems able to sidestep the above-mentioned problems. Recently, deep neural networks (DNN) and their associated learning paradigm, deep learning (DL), have offered spectacular results in many signal processing applications. For our problem, the usage of DL/DNN in the computer vision (image and video) domain is of particular interest. 

Our aim in this article is to review and compare the most recent deep learning-based solutions, along with employed sensors, datasets and performance metrics used for the PTP problem.

The key contributions of this article are summarized as follows.

- The *sensor types*, typically used in the context of our problem, are presented in relation to the DL techniques that use them.
- A detailed analysis of *deep learning approaches* for pedestrian trajectory prediction is performed.
- An overview of the most important *datasets* in the field, considering sensor types, size, number of pedestrians, location, view, etc., is also provided.
- An emphasis on the *research gaps* from the literature and an outline of potential *new research directions*.

The rest of this article is organized as follows.

Section 2 presents a review on sensor technologies used for ADAS applications, in general, and for the pedestrian trajectory prediction problem.

Section 3 links the sensorial information to the deep learning paradigm, by investigating DNN approaches for PTP.

Section 4 addresses the datasets used for evaluation, along with useful metrics, and the reported experimental results.

Section 5 concludes the paper.

## 2. Sensor Technologies for Pedestrian Trajectory Prediction

The following sensor types are often employed in the problem of PTP: radio detection and ranging (radar), light detection and ranging (LiDAR) and video camera.

Parallel to sensor development, new techniques based on sensorial information fusion emerged. Key aspects of those technologies are briefly presented below. 

### 2.1. Automotive Sensing

A self-driving vehicle (SDV) is a vehicle retrofitted with sensors and systems able to automatically control the vehicle such that it can run on the road without human intervention. To be able to perform such tasks, SDVs rely on several sensors (such as LiDAR, radars, and cameras) to detect objects in its vicinity, as well as predict their future trajectory and their motion uncertainty [7]. The sensors and the systems processing their information also assist the drivers by signaling different circumstances to minimize the risk of exposure or by collaboratively automating driving assignments in order to reduce human errors [8]. Usually, the measurements referring to internal sensors (known as “proprioceptive”) are not enough to deliver warning and safety applications related to the external environment.

Regarding exteroceptive sensors, SDVs acquire environment information. Processing the received information leads to recognizing other factors and objects located nearby. In recent years, SDV external sensing has gained importance, especially with camera and image processing development, since these systems enable a wide range of applications [9]. 

**Figure 1:**
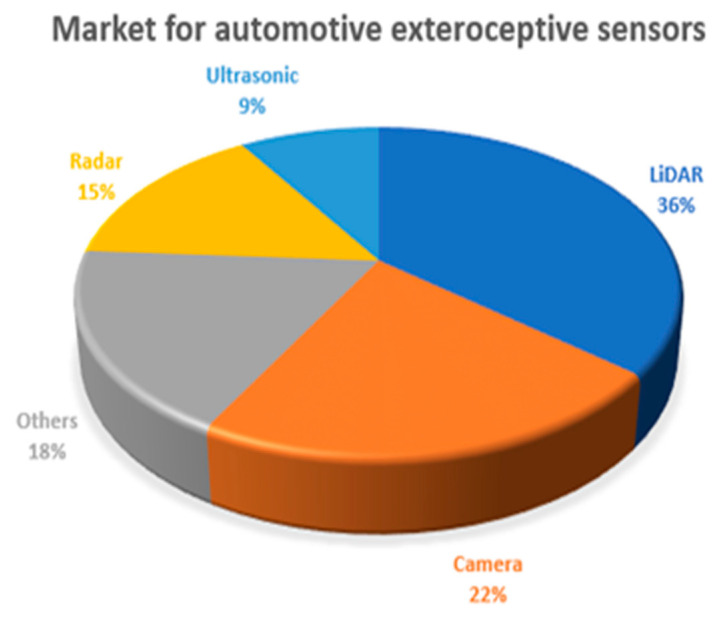
The prediction of automotive sensors market growth (compound annual growth rate (CAGR), 2017–2022) on exteroceptive sensors.

As stated in [10], autonomous driving assistance is based mainly on systems related to image and camera processing. LiDAR represents the most required sensors in automotive systems. In contrast to cameras, it is characterized by omnidirectional sensing, and it is not affected by light conditions. One quarter of the market is represented by ultrasonic and radar sensors, while other exteroceptive sensors (e.g., microphones) accumulate 18% of the market (see Figure 1).

### 2.2. Radar

The primary goals of automotive radar systems are to determine the targets of interest (e.g., pedestrians, cars, or cyclists) and estimate their size, motion, distance, relative velocity, and direction regarding the radar [11]. Using reflected electromagnetic waves, which are received and transmitted simultaneously, radar monitors the entire image of the environment. Up to the present, because of some inconvenience, radar data were used only in few cases of pedestrian trajectory prediction.

Radar systems involve the transmission of radio wave pulses, which can bounce on targets in front of the vehicles. A problem was observed referring to the interference of different information due to reflected pulses arriving later at the sensor. 

The frequency changes due to Doppler shift can facilitate the measurement of the relative speed of moving objects (e.g., pedestrians). Automotive radar systems commonly perform at frequency bands of 24, 79, and 77 GHz (for the most recent radar generations), and it can cover angles between 9° and 150° [12]. Radar can operate in unfavorable conditions (e.g., rain, dust, snow, or fog) [13] with three distance ranges: long range (10–250 m), medium range (1–100 m) and short range (0.15–30 m). Regarding range, distance estimation has an important role. In order to determine the range, the round-trip time delay is used to characterize the electromagnetic waves which need to circulate to and from the target.

**Figure 2:**
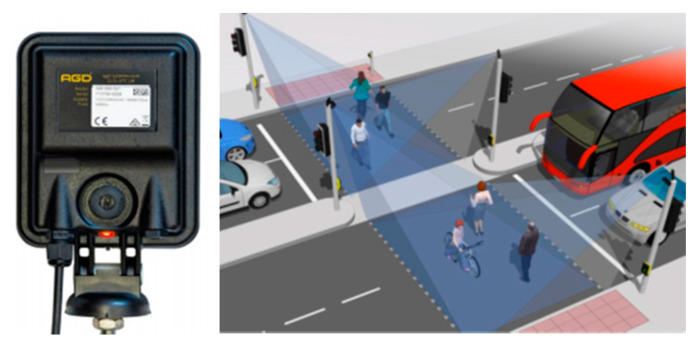
The AGD326 radar is a 24 GHz pedestrian detector can be used for crossing phase optimization. (Image source: www.agd-systems.com; accessed on 30 October 2021).

A key issue in the prediction of the pedestrian trajectory is represented by the estimation of the target velocity and the distance between pedestrians and the sensor (see Figure 2). To ensure a controlled direction of the emitted wave and the distinction of targets based on the velocity and distance, the last models of automotive radars use frequency-modulated continuous wave (FMCW) technology along with digital beamforming [14]. The direction estimation relies on obtaining data regarding the reflected waves throughout numerous different dimensions, which are obtained by combining the frequency, space, and time variables. For example, in [15], a method was proposed to learn and predict the dynamics of moving targets (e.g., pedestrians) by applying the data measured by the fast chirp FMCW resolution radar directly to the LSTM and CNN models. In the proposed architecture, the time-series radar data were measured and transformed into range-velocity images by two-dimensional discrete Fourier transform. They tested the proposed method using the data obtained by fast chirp FMCW radar of Hyundai Mobis.

The automotive radar mechanism is influenced by some unwanted reflected information along with the reflected waves from targets of interest. This adverse amount of data, in the form of noise or clutter, which is a reflection from the walls, road trash or guard rails, leads to an alteration of the surrounding environment. Consequently, there were developed different adaptive algorithms to attenuate the effect of these perturbations. Space-time adaptive processing (STAP) [16], as well as constant false alarm rate (CFAR) [17,18] represent two important algorithms, which can be used in this issue. For an accurate detection of the target of interest in the presence of noise, the value of the threshold should be considered, which must be correctly established, depending on the amount of noise in the system (as the noise expands, a higher threshold must be established). Ding et al. [19] proposed a technique to extract pedestrian micro-Doppler trajectories from continuous-wave radar echo. They used CFAR to estimate, in real time, the noise parameters and adjust the filter threshold. After denoising, fake pedestrian detection can be greatly suppressed and some signals are more easily detected. For denoising, the authors used the “CLEAN” algorithm [20], where multiple components from the continuous-wave radar echo are extracted sequentially, each parameter is estimated, and the stronger components are removed.

It can be defined as a micro-Doppler effect, essentially a considerable small Doppler shift, which was obtained because of the micromotion that characterizes a pedestrian [21]. Another important concept is the micro-Doppler signature, which is the periodic pattern followed by velocity in time due to the periodic movement of limbs. In order to specifically detect pedestrian walking, there are also different algorithms used, which may include extraction and matching. In [22], the authors developed a method using a combination of automotive and Doppler radars to detect the motion components of pedestrians by applying Gaussian distribution and a Kalman filter. By analyzing the Fourier spectrogram of Doppler frequency, they can detect the motion of humans in periodically. To test the method, they collected four data sets from different environments. Another work was proposed in [23] to predict pedestrian movement behaviors. They used simultaneous radar measurements and motion capture sensors for digital recording of movements for each individual body part. For detection, the authors used a CFAR algorithm where each range doppler cell is estimated by a detection threshold. The results and characteristic features of movement behavior are provided only from one pedestrian. 

Dubey et al. [24] presented a Bayesian framework to integrate motion and appearance modalities of pedestrians into the tracker. To distinguish and learn features for each class, they created a distance metric learning over a latent feature vector. Pedestrian trajectories were interpolated from individual waypoints with constant velocities by combining tracking and classification systems. To generate different scenarios, the authors used MATLAB driving scenario designer. The authors of [25] presented a method for estimating pedestrian motion direction in complex scenarios using their micro-Doppler signature obtained by the automotive MIMO radar. This method observes the pedestrians from a single angle and extracts the motion direction information from the micro-Doppler signature by using regression methods. To test the proposed method, the authors used automotive scenario simulations, where the pedestrian is observed in multiple input/output radar sensors.

As autonomous driving assistance technology is growing in the market, automotive radar is taking major steps toward becoming a more powerful solution for the problem of pedestrian trajectory prediction. This transformation involves all aspects of automotive radar, including system concept, modulation, and signal processing.

### 2.3. LiDAR

**Figure 3:**
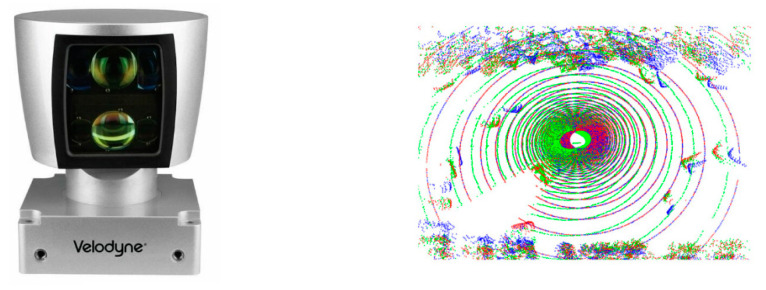
A 3D-LiDAR sensor could be employed for short, medium, telescopic or combinations (dual short range, dual medium) range. Here, a Velodyne HDL-64E sensor and its generated points cloud. (Images source: www.velodynelidar.com; accessed on 29 October 2021).

There were developed LiDAR (light detection and ranging) sensors, which are based on laser reflection for the detection of objects surrounding the vehicle. It is known that LiDAR sensors emit periodical light pulses, e.g., every 30 ns. The light beam transmitted by the sensor has a typical wavelength of 905 nm, and it is coaxial with the reflected light component [26]. The LiDAR technology has a great accuracy due to the circular and vertical way of action, which allows to obtain 3D models—spatial illustrations of coordinates acquired by recording the distance and the direction of the returning light pulses as data points, which afterward, are organized into point clouds. LiDAR sensors facilitate an innovating collection of trajectory-level data in case of mixed traffic conditions. Using 3D point clouds [27], these sensors are capable of reporting the precise location of the objects and may cover angles of up to 42° the vertical range of visibility and at 360° the horizontal range surrounding the vehicle, without being influenced by light conditions (see Figure 3). An important disadvantage of LiDAR technology is the small sensitivity to light and atmospheric conditions. It can be said that LiDAR sensors are not appropriate for real time implementations because they are considered to be time-consuming. LiDAR sensors that have a small price (starting from USD 100) are characterized by a single light beam and small power usage (starting from 8 W). Meanwhile, the newest models of LiDAR sensors have a better point cloud resolution, utilizing laser arrays (up to 128).

Compared to other sensors, for instance digital cameras, LiDAR sensors can lead to a better perception in all illumination conditions, which make it remarkable in the autonomous-driving vehicle. Although the data’s accuracy is reduced by unfavorable weather conditions, such as rain or fog, under moderate weather conditions, the LiDAR sensor can be properly used in high-frequency applications (e.g., creating a perception layer in case of an autonomous vehicle). The high-class LiDAR could work in all illumination conditions, being able to generate exhaustive local maps for an ego vehicle. These maps may be useful in behavior predictions, in regard to the surrounding environment and vehicles. More exactly, the environmental behavior predictions have a critical role in the predictive path planning of a self-driving vehicle; for instance, the possibility of making a turn of a certain vehicle ahead can be predicted.

**Figure 4:**
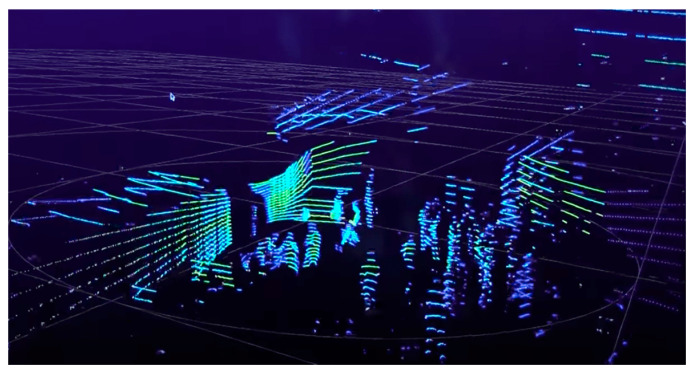
Raw LiDAR data of pedestrians, captured using Velodyne HDL sensor. (Images source: www.velodynelidar.com; accessed on 29 October 2021).

LiDAR sensors are currently used mainly for the detection of obstacles, road users and lane markers in autonomous vehicles [28,29,30,31]. Using intense point clouds, on-board LiDAR sensors are able to create an exhaustive description of objects, while roadside LiDAR sensors provide sparse data points. Figure 4 shows one example of the raw LiDAR point clouds detection of pedestrians. The characteristic of data depends on the distance between LiDAR and the pedestrian.

The depth information provided by LiDAR was directly used by several researchers to cluster points, estimating the pedestrian’s location as a 3D bounding box. The vertical and horizontal angular resolution that characterizes this type of sensor influences the point clouds’ density. 

**Figure 5:**
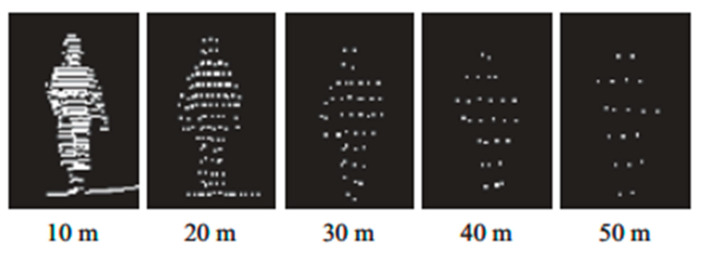
Pedestrian 3D point clouds characteristics at different distances.

Figure 5 shows the shape of point clouds from 3D LiDAR scans of pedestrians at different distances between the pedestrian and the sensor. The XYZ projections are calculated from a sample that has enough points cloud.

In [32], the authors developed a complete roadside LiDAR data processing system using direct row data in a 3D Cartesian coordinate system from sensors to predict future trajectories of pedestrians in real time. The extracted future trajectory had information about the XYZ position, the total number of data points, the distance between LiDAR and the pedestrian, velocity, tracking ID, timestamp, frame number and the label of each pedestrian. They used a VLP-16 LiDAR sensor model from Velodyne Lidar company, San Jose, CA, USA, with 16 lasers rotated horizontally and an internal motor in the XYZ coordinates. To classify the sequence data from the sensor, such as trajectories of feature-based classifications, the author used the Naïve Bayes algorithm [33] applied at the input of the model for different ranges for probability calculation and an optimal combination of features.

**Figure 6:**
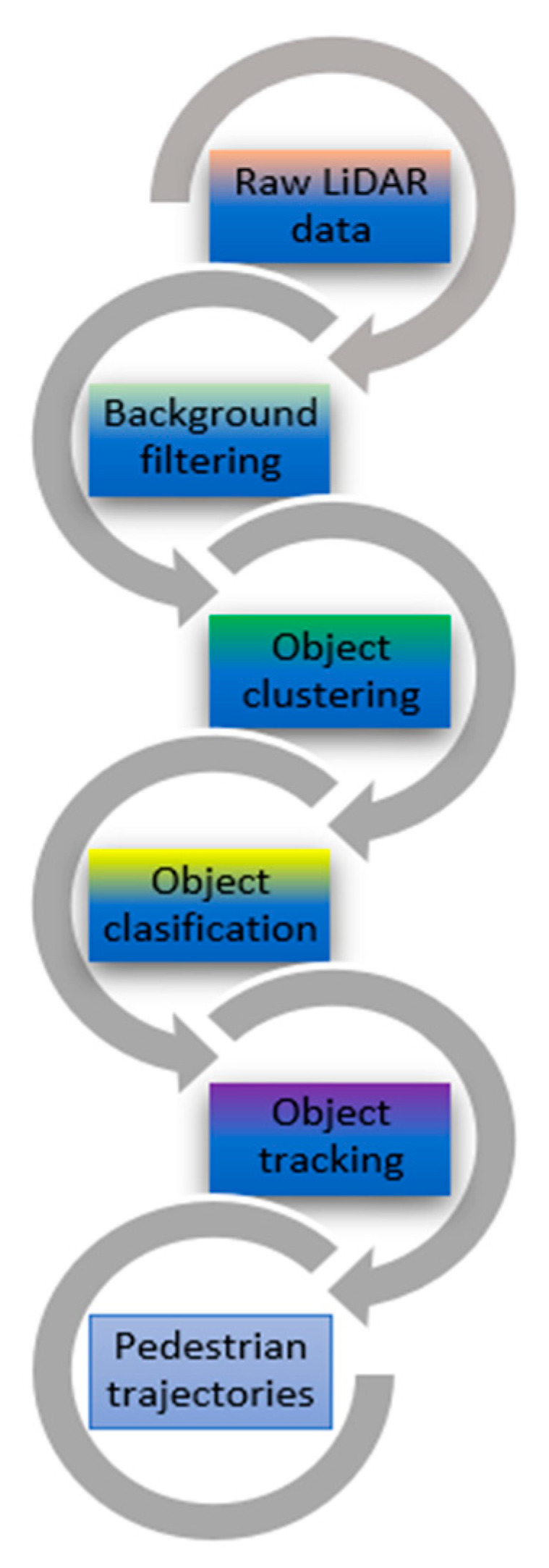
Flow chart of data preprocessing.

To extract the trajectories of pedestrians from raw LiDAR data, a data preprocessing procedure is needed to perform background filtering [34], object clustering [35], object classification [36] and object tracking [37,38], as depicted in Figure 6. 

Bu et al. [39] proposed a method that can perform 3D-oriented pedestrian estimation based on 2D LiDAR data and monocular camera. This method consists of three sub-networks (orientation network, regional proposal network and PredictorNet) to perform more accurate predictions with bounding boxes. The orientation network reseizes and crops the data to determine the orientation angles. The region proposal network takes feature maps and inputs from the orientation network and generates non-oriented pedestrian bounding boxes. PredictorNet uses the pedestrian feature map obtained from previous networks to make a final prediction and classification.

In [40], Völz et al. presented different architectures that can be evaluated to identify pedestrian intentions to cross the street at a given crosswalk. They introduced a dense neural network architecture to classify pedestrian intentions based on features from several timesteps, reaching the cross-validation accuracy at 96.21%. To more accurately analyze the time-series features, they used recurrent neural networks, which allowed feeding data back into the dense neural networks, reaching a cross-validation accuracy of 95.77%. Using convolutional neural networks, image features are extracted by proposed convolving trained filters along the image from LiDAR, and those features are used to classify the data. To implement these architectures in Python, the authors used Theano [41] and Lasagne [42] DNN implementation frameworks.

An interesting solution for online estimation of pedestrian position, velocity and acceleration was proposed by Mohammadbagher et al. [43]. To identify the pedestrians in the image captured from the LiDAR, they used a deep neural network architecture based on object detection (YOLOv3 Pytorch). To localize the ego vehicle and hence the pedestrian of interest in the image, they used the odometry information captured from GPS/IMU sensors. The authors tested the model in two experiments and different image scenarios. 

While roadside LiDAR sensors are able to perform independently, on-board LiDAR sensors need other sensors, for instance radar or cameras, in order to support the systems in autonomous driving. The high costs together with the limited applications in regard to the implementation of the roadside LiDAR sensors are responsible for its limited usage, even if this type of sensor can provide trajectory-level and a real-time data collection. In [44], the authors proposed a subsystem to handle pedestrians in crosswalks by applying deep learning methods directly to the data from a fusion between the camera and LiDAR sensors. To detect the pedestrian, the authors used CNN and the data from the camera; to find the positions of pedestrians in images they used the data from LiDAR point cloud. 

However, a wide deployment of LiDAR sensors will soon be possible due to an increasingly expanded application market, along with the recent progress in LiDAR technology and the newly publicly available data (e.g., nuScenes prediction challenge and Lyft Motion Prediction for Autonomous Vehicles). Considering the fact that roadside LiDAR technology cannot directly use the methods applied for on-board LiDAR data processing, it is crucial to analyze the basics of roadside LiDAR, including the installation strategies and the effective and efficient techniques regarding online and offline data processing.

### 2.4. Video Camera

**Figure 7:**
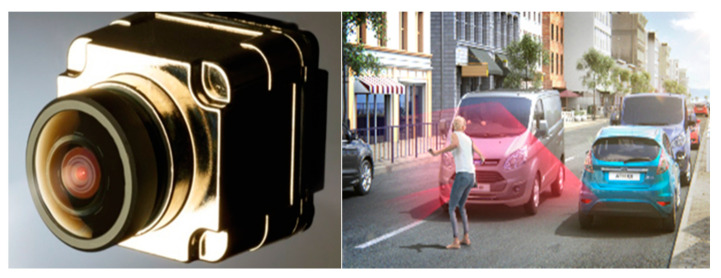
Pedestrian detection using SVC cameras. A Valeo 360 surround view camera could offer a three-dimensional view of the environment. (Image source: www.valeo.com/en/360-vue/ and www.fordclubsweden.se; accessed on 12 March 2021).

The purpose for the vision sensor is multifarious: it could be used for looking both inside, for driver and occupant monitoring, and outside, for object (traffic lights, road signs, other traffic participants) detection [45]. New concepts are represented by inside–outside information fusion and the surround view camera (SVC) system [46], consisting of four cameras—in front, in the rear, and on the outside rearview mirrors, and it is able, among other tasks, to recognize nearby pedestrians early (see Figure 7).

The CMOS-based cameras could operate in multiple spectral bands, e.g., visible (VIS), near-infrared (NIR) or short/long-wave infrared, each of them offering useful features in various traffic scenarios (day, night, fog, snow, etc.). They could be further classified according to their resolution, field of view, or number of video cameras (mono, stereo vision, SVC).

The main advantages of this kind of sensor are the reduced price and low power consumption, whereas the disadvantages are related to the performance dependability of light/traffic conditions.

Despite the increased amount of the provided information, there is an increasing interest in analyzing actor (humans, vehicles) behavior from *video* data provided by this kind of sensor. In this context, the problem of trajectory/path prediction is mainly presented in the literature in two distinct situations: (1) when the video data is provided by a surveillance—terrestrial or aerial—system’s cameras and (2) when the input comes from the sensorial system of an automobile. The first approach addresses mainly security applications, whereas the former concerns the active safety of a car. Nevertheless, the functioning principles presented in these two cases are interchangeable, i.e., a method used in path prediction for a surveillance system might be useful for an automotive application. The future location prediction from camera could also be performed from multiple points of view: close or far shots and first-person or third-person perspective. Of particular interest for our presentation is the case of pedestrian motion trajectory prediction from far shot first-person perspective [47].

Most of the early approaches used classical/statistical paradigms and hand-crafted dynamic functions to estimate the risk of collision between the vehicle and pedestrian. A typical case is the use of Kalman filtering [48] for vulnerable road users. Such an example is the work of Keller et al. in which stopping motion is detected using two Kalman filtering approaches, versus two stereovision-based methods using dense optical flow [49]. A pedestrian trajectory destination identification based on raw videos is presented in [50]. It is based on statistical approaches (Gaussian mixture models/background subtraction for segmentation, Otsu’s thresholding, silhouette, and star skeleton extraction) in which motion features such as position, velocity and acceleration are calculated from the human skeleton. For the final step of pedestrian destination prediction, several models were tested, e.g., multinomial logistic regression (MLR) and multi-layer perceptron (MLP) ensemble. The support vector machine (SVM) provided the best median AUC value of 87%.

Prior works considered forecasting trajectories using a single camera view. There are other approaches that forecast pedestrian trajectory based on multiple non-overlapping camera views, e.g., the work of [51]. Here, multi-camera trajectory forecasting (MCTF) is performed using multiple baselines such as shortest real-world distance or most similar trajectory. A long short-term memory (LSTM) and a gated recurrent unit (GRU), both with 128 hidden units, yield the best results: 74.4% and 75.1% respectively, top accuracy using Warwick–NTU multi-camera forecasting database (WNMF).

Today, for camera images, deep learning has become the state-of-the-art method for both 2D- and 3D-type of data, as summarized below.

Most camera-based approaches are formulated as ego-motion forecasting, e.g., [52], where the problem of ego-vehicle trajectory is solved via semantic segmentation of the data provided by a single monocular camera. The authors propose an end-to-end sequence-based network based on FlowNet [53], AtrousCNN [54] and Spatial Pyramid Pooling (ASPP) from Deeplab [55] and obtained, using KITTI dataset [56], an 89.00% accuracy and 72.25% IoU for a 5 s prediction horizon.

Loukkal et al. [57] stressed the practical importance of a system in which only monocular cameras are used. They proposed a two-stage deep neural network-based architecture in which a mapping from camera image to a bird-eye-view occupancy grid map was first performed. Then, a second stage performs motion planning, using a LSTM encoder–decoder configuration. The reported results show an ADE of 0.78 for the holistic end-to-end model versus the nuScenes dataset [58].

Other approaches performing future person location and movement trajectory prediction using vehicle-mounted cameras are shown in [59,60]. Conversely, wearable cameras were employed in [61,62]. The authors from [62] described an LSTM-based encoder–decoder system which uses locations and poses of the targeted person and inertial measurement unit (IMU) data from an egocentric wearable camera (GoPro Hero 7 Black) as the input and is able to predict the future location and movement trajectory of the targeted person.

In [63], the authors make an important observation: it “is much more efficient to learn and predict pedestrian trajectories in the 3D space since the human motion occurs in the 3D physical world”. Their solution used a stereo camera for providing 3D information. From it, they extracted, using a twin poseGAN—DNN, a pose estimation. The solution could be seen as an extension of the Social GAN from 2D into the 3D domain.

### 2.5. Comparative Features of Sensors

The newest trends enhance the image processing capability of a car by mixing the information from multiple automotive sensors. For example, in the work of Meyer and Kusch [64], Astyx 6455 HiRes radar sensor, Point Grey Blackfly camera and a Velodyne VLP-16 lidar were used as inputs for CNN-based low-level sensorial information fusion. Zhang et al. further proposed a vehicle-mounted sensing system collection containing a Velodyne HDL-32E lidar, an inertial navigation system (OxTs Inertial + GNSS/INS suite) and a Mako camera used for prediction of pedestrian risky level [65]. An LSTM network was employed to predict the trajectory of pedestrians based on short durations (3.23 s on average) and used 36 pedestrians to collect trajectories. They reported an average displacement error (ADE) of 0.5074 m. For risky level classification, the authors proposed a combination of K-means clustering (KMC), kernel principal component analysis (KPCA) and a kernel support vector machine (SVM). To know where these automotive sensors can be utilized, in report [66], a retrospective of each automotive sensor based on performance aspect was presented (see Table 1).

## 3. Deep Learning Paradigms for Pedestrian Trajectory Prediction

**Figure 8:**
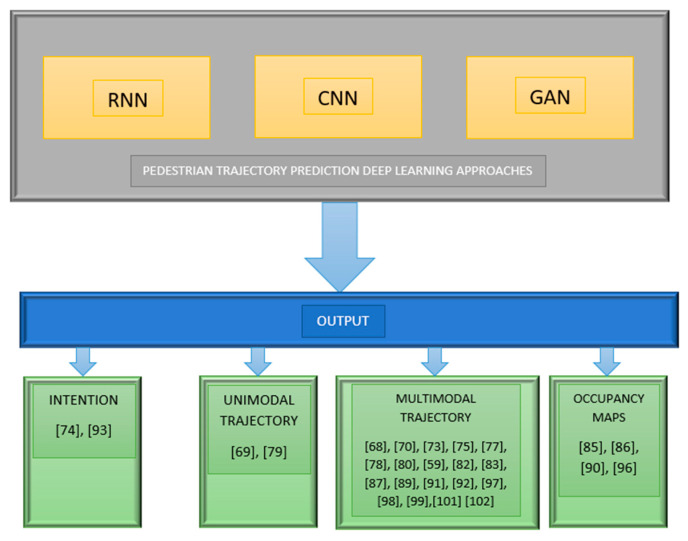
PTP DL-based techniques to references mapping.

To solve the problem of PTP, in the last years, several deep learning-based methods have been proposed in the related literature. This section details the most utilized methods from this area, classified according to the DNN architectural type. The identified pedestrian trajectory prediction deep learning-based methods used mostly three architectural structures, as follows. See also Figure 8 for a systematic mapping between the surveyed techniques and the corresponding references.

- Recurrent neural networks (RNN), typically in the form of long short-term memory (LSTM).
- Convolutional neural networks (CNN).
- Generative adversarial networks (GAN).

In many situations, the proposed systems mix the above-mentioned DNN types. 

### 3.1. Trajectory Prediction Based on RNNs

**Figure 9:**
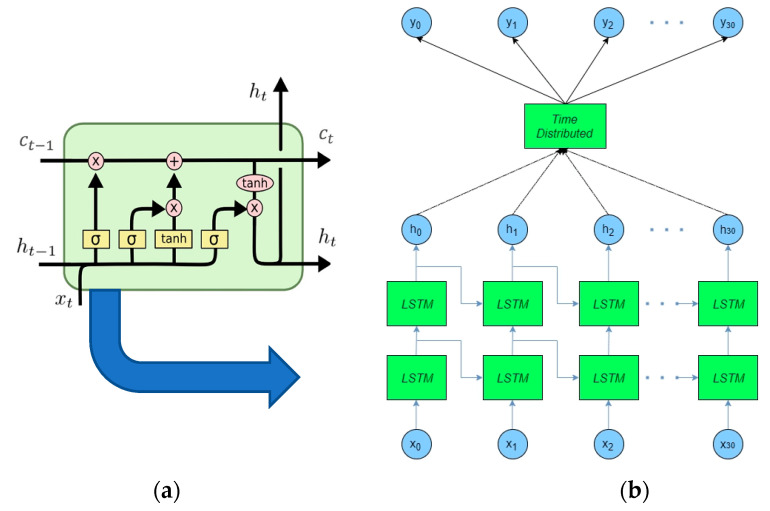
(**a**) LSTM cell. (**b**) Deep RNN using LSTM.

A recurrent neural network known as Vanilla RNN is an extension of a two-layer fully connected neural network where the hidden layer has a feedback loop. This small change allows to model sequential data more efficiently. The Vanilla RNN works not only with the input data belonging to the current step, but also with data of the past steps, which is stored in the anterior hidden neurons. RNNs have an important role in the sequence-relied prediction, which is used in many applications, as it is explained in Figure 9.

The challenge to cope with long-term information preservation has been successfully addressed using a long short-term memory (LSTM) structure [67]. Demonstrating the first good results in a natural language processing (NLP) domain by modeling latent data features, the LSTM is also used for pedestrian trajectory prediction. For example, in [68], Sun et al. used the LSTM model to learn the environment and people activity patterns in the target environment from long-term observations (i.e., several days to several weeks). 

In order to predict the human body pose, in a system based on motion capture and also in videos, Fragkiadaki et al. [69] showed a method that relies on recurrent neural networks, using encoder–recurrent–decoder (ERD) architecture. ERD architecture is an extension of the long short-term memory (LSTM) model that incorporates nonlinear encoder and decoder networks before and after recurrent layers. The encoder transforms the input data to a representation, and the decoder transcribes the output of the recurrent layers to the desired visual form. In this way, the proposed architecture can predict the future position of pedestrians by analyzing the whole-body position. 

Alahi et al. [70] proposed a social LSTM model to predict joint trajectories in continuous spaces. Considering the fact that neighboring people have influence on humans, LSTMs share the information stored in the hidden state with the nearby pedestrians, creating a social pooling system. Their model is reported to outperform state-of-the-art methods on several datasets. In their work, they employed one LSTM model for each trajectory, therefore called “social LSTM”. This model was tested on ETH [71] and UCY [72]. A brief overview of the results is presented in Table 2.

S. Dai et al. [73] proposed a spatial–temporal trajectory prediction model based on LSTM. According to them, LSTM networks cannot simultaneously describe the spatial interactions between different vehicles. In addition, they underlined the fact that the LSTM models suffer from a gradient vanishing problem. They introduced a connection between the input and output of two consecutive layers to handle the gradient vanishment and solve the trajectory prediction in dense traffic. The proposed model’s performance was tested on I-80 and US-101 datasets. Their model was reported to deliver trajectory prediction with higher accuracy than in other state-of-the-art models. 

L. Xin et al. [74] were concerned with what they called “long-horizon trajectory prediction of surrounding vehicles”. Their method (a deep neural network architecture based on intention-aware LSTM) is reported to learn high-level and spatial–temporal features of driver behavior. Their network was trained on the NGSIM dataset. Their test results on highway data show a more accurate estimate in comparison to other methods. Longitudinal and lateral prediction are less than 5.77 and 0.49 m, respectively.

In [75], Lee et al. proposed a trajectory prediction framework named deep stochastic inverse-optimal-control RNN encoder (DESIRE), which works for various interacting agents using deep neural networks. In order to generate hypothetical future trajectories, a conditional variational auto-encoder was used. Afterward, an RNN model was used to rank and score these characteristics based on an inverse optimal control mode, taking into account the scene context. This method accounts for the multi-modal nature of the prediction and estimates the potential future outcome. A feedback algorithm was also used for boosting the estimation accuracy. The model’s performance was evaluated using the KITTI [56] and the Stanford Drone Dataset [76].

A hierarchical policy method was developed by Zheng et al. [77]. This approach automatically reasoned about short-term and long-term goals. The solution is based on recurrent convolutional neural networks to predict both the micro-actions (relative motion), and macro-goals (intermediate goals). These DNNs were trained individually using supervised learning, together with an attention module and, ultimately, were jointly fine-tuned. This method was extended by Zhan et al. [78] using variational RNNs.

Martinez et al. [79] described an approach, which relies on RNN with gated recurrent unit (GRU) architecture, permitting to train a single model on the entire human body without the need of a spatial encoding layer. Instead of working with human absolute angles, they modeled velocities. They proposed what they called “residual architecture”, which models first-order motion derivatives. An increased accuracy and smooth prediction were reported. 

Hug et al. [80] proposed an LSTM with a mixture density layer (MDL) model combined with a particle filter method for multi-model pedestrian trajectory prediction. Their implementation used vectorized calculations, and it was implemented in TensorFlow. Their model was tested on several intersections of “T” shape. The authors used scenes from the Stanford Drone Dataset in their experiments. Scenarios where maximum likelihood predictors would fail due to their inability to deliver multiple hypothesis were used. Such scenarios include roundabout-type intersections. 

A long-term prediction model using RNNs was proposed in [59]. The encoder–decoder architecture jointly predicts the ego motion as well as people trajectories. The authors claimed that by using the model, one can predict human trajectories at desired time horizons. Training and performance assessment were performed using the Cityscape dataset [81]. 

In [82], T. Salzmann et al. proposed a method that forecast the futures–conditional trajectories of the general number of agents (i.e., pedestrians, vehicles) with distinct semantic classes, while including heterogeneous data. To encode agent interactions, they developed a system in which each agent has a semantic class namely car, bus or pedestrian and provides information about their position histories with context size, spatial resolution, and semantic channels. The authors tested their model on the ETH, UCY and nuScenes datasets [58]. A brief overview of the results is presented in Table 2.

Xue et al. [83] present a hierarchical LSTM-based network for pedestrian trajectory prediction in crowded scenes. They started from the idea of pedestrians walking in a crowded place, some obstacles such as neighboring pedestrians and scene layouts can affect their moving trajectories. Three LSTM encoders were used for three scales: person (capture each individual trajectory information), social (capture information about neighborhood) and scene (capture information about layouts). To test the method, they used ETH, UCY, and Town center Dataset [84].

### 3.2. Trajectory Prediction Based on Convolutional Neural Networks

**Figure 10:**
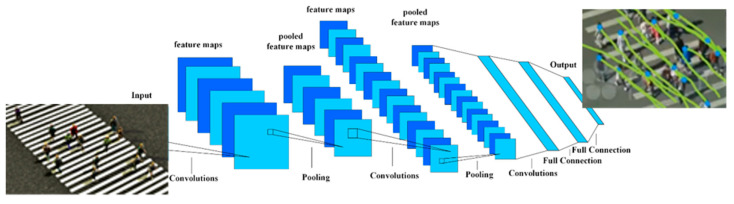
CNN-typical architecture: input, multiple convolutional + ReLU activation function, (max) pooling, flatten, fully connected and SoftMax outputs layers.

The convolutional neural network (CNN) represents a DNNs type, which has a great performance in many fields, such as object classification and recognition, e.g., handwritten numerals, letters, and faces. CNN has the typical architecture presented in Figure 10, and it contains a large number of convolutional, non-linearity, pooling, dropout, batch normalization, and fully connected layers. As a result of the network training/optimization, CNNs are able to learn object features. The suitable choice of network architecture and parameters makes these features include the most significant discriminative information required for the robust identification of the targeted objects.

Rehder et al. [85] proposed a method to infer pedestrian future destinations from images and position using a recurrent mixture density network. In order to develop a trajectory prediction such as goal-oriented motion planning, there are two different architectures used: a forward–backward network and an MDP (Markov decision process) network. The image and the position of pedestrians serve as an input for the architecture. The processing of the image is performed via a CNN network. The concatenation of the position vector and of CNN output represents the input to a LSTM network. As output, the network makes a prediction of probable future destinations of the pedestrians using a probability distribution map. To train and evaluate the proposed network in the real world, the authors collected stereo videos with manually pedestrian annotations from multiple drives through urban and residential areas. 

In [86], S. Hoermann et al. proposed a method that combines two networks: a CNN for long-term motion and a Bayesian estimate of the current dynamic environment as input. The analysis of the scenes is based on a 360° predicting area in a single neural network besides the network that performs the segmentation of the static and dynamic areas. Using rare dynamic cells, the authors created a loss function based on the counteracting of imbalanced pixels from various categories. They proved the capacity of the network to predict highly complex scenarios with different road users (i.e., pedestrians) of various types for up to 3 s. In addition, the network can identify different maneuver classes, e.g., turn left or right, and interactions between road users.

Zhao et al. [87] proposed the multi-agent tensor fusion (MATF) network with encoder–decoder architecture. The spatial centric approach uses a flexible network that can be trained in contextual images of the environment with sequential trajectories of agents, retaining spatial relations between features and capturing interactions between the agents. Their model encodes the past trajectories of each individual agent independently and decodes recurrently to multiple agents’ futures trajectories, using adversarial loss to learn stochastic predictions. They trained the method on the ETH-UCY dataset, Stanford Drone Dataset, and NGSIM Dataset [88]. See Table 2 for details.

In [89], Yi et al. proposed the behavior-CNN model that is trained with crowded scenes video data. A pedestrian behavior model was developed to predict their future of walking path and destination. This model can deduce scene frames from motion characteristics in input and the learned location bias map. To increase the tracking accuracy of pedestrians, the model can provide important information based on predicted pedestrian walking paths.

Doellinger et al. [90] used CNN to predict average occupancy maps of walking humans even in environments where information about trajectory is not available. Their method is reported to perform better than several baseline methods. They employed a mobile robot to record images and create a dataset of their own. Their work has demonstrated that human occupancy distributions can be used to find waiting positions.

In [91], Marchetti et al. presented MANTRA, which is a model based on memory augmented neural networks (MANN), which studies the connection between past and future pedestrian motion and memories in the most significant samples. MANTRA is capable of updating internal representation of motion samples in online learning. Therefore, as new sample are collected, the model improves. The authors conducted their testing research on three available traffic datasets: KITTI dataset [56], Oxford RobotCar dataset, and Cityscapes dataset. See Table 2 for more details.

Mohamed et al. [92] proposed an approach by modeling the interactions between pedestrians as a graph representation used to extract meaningful features. These features have information about the compact representation of the observed pedestrian trajectory history. To predict the future trajectories of all pedestrians, the authors created a second layer (time–extrapolator CNN) with input T × P × N (P—dimension of pedestrian position, N—number of pedestrians, T—number of time steps). TXP–CNN works directly on the temporal dimension of the graph embedding and expands it as necessary for prediction. To evaluate the method, the authors used the ETH and UCY dataset.

Wang et al. [93] presented a method that refers to the analyzation of the spatial interactions between different objects and backgrounds from the scene regarding the trajectory prediction of pedestrians. They combined human pose estimation and 2D–3D size information of the pedestrians into the model to predict their intentions. They adopted the monocular image depth estimation method to instantly extract the depth map of the image around the pedestrian at each time and to manipulate the model in order to learn the human–object interaction and the human–scene interaction. The authors set a bottom center of the 2D bounding box for pedestrians to identify the location of these in the image. To evaluate the method, MOT16 and MOT20 datasets were employed [94].

### 3.3. Trajectory Prediction Based on GAN

**Figure 11:**
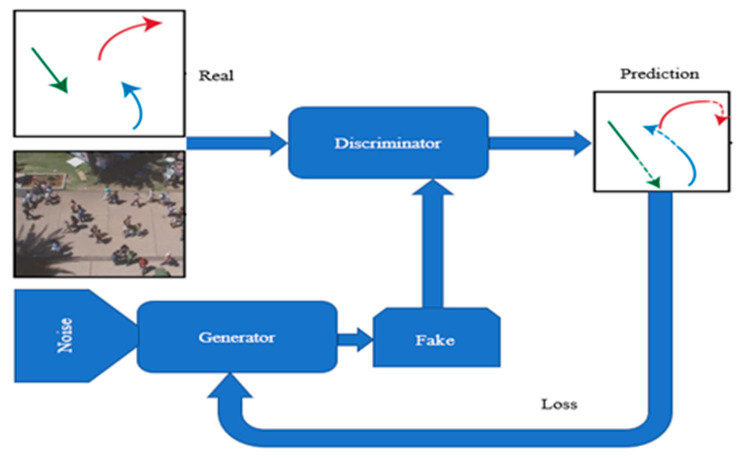
A typical GAN architecture for trajectory prediction.

Generative adversarial networks (GAN) [95] rely on generator (G)–discriminator (D) architecture. They compete against each other: the G network attempts to fool the discriminator network, whereas the D network adapts to the new fake data. Thus, in a GAN framework, a generator model and a discriminator model are trained simultaneously (Figure 11).

In regard to tracking, GANs reduce the fragmentation that usually appears in many conventional trajectory prediction models and mitigate the necessity to compute expensive appearance features. The candidate observations are produced and updated by a generative component; afterward, the least updated are eliminated. In order to process and classify the candidate sequences, there is, concomitantly used, a LSTM component with a generative–discriminative model. This method can lead to high-accuracy models of human behavior, particularly group behavior. Conversely, compared to the previously known CNN-based solutions, it is considerably more lightweight. Recently, many authors have applied the GAN architecture to achieve multi-modality in the prediction output, as explained below.

In the work of [96], each input frame was passed through a GAN generator, which in response, output a probability map for each pixel. This map was further watershed segmented. The prediction was made from both short (used for data association) and long (used to update the trajectory of the objects) term perspectives. The person detection stage was evaluated on PETS (performance evaluation of tracking and surveillance) S1L2 and S2L1 datasets by reporting multiple object detection accuracy (MODA) and multiple object detection precision (MODP), precision and recall. The tracker evaluation was performed on 3D MOT (Multi Object Tracking) 2015 and ETH Mobile Scene (ETHMS) dataset benchmarks having multiple object tracking accuracy (MOTA), multiple object tracking precision (MOTP) and mostly tracked targets (MT) as notable metrics.

A socially aware GAN model with RNNs was proposed by Gupta et al. [97] for pedestrian motion prediction in multiple and dynamic environments. They started from the idea that pedestrians influence each other uniformly, while including the impact of all agents in the scene, likewise as the scene context. Socially plausible futures were predicted by training adversaries against a recurrent discriminator. An encoder–decoder architecture was used. A novel pooling mechanism was used to aggregate information. The used datasets were the ETH and UCY. As metrics, they employed average displacement error (ADE) and final displacement error (FDE), with an evaluation methodology similar to [70], for 8 (3:2 s) and 12 (4.8 s) time steps.

Kosaraju et al. [98] followed the same idea of social interactions found in [96,97] plus scene context and the multimodal behavior of pedestrians in proposing a graph attention network that encodes these factors. Further, GAN architecture will forecast human paths. To test this solution, the authors used ETH and UCY datasets because these datasets contain annotated information about pedestrian trajectories and interacting pedestrians in public scenes.

Amirian et al. [99] proposed a method that relies on Info-GAN [100] for the prediction of pedestrian trajectories within a time interval of several seconds in the future. The traditional L2 loss term was replaced by an entropy-based cost function, due to the negative impact over the network generalization capability. See details of this GAN advanced training method in [101]. The results were reported against the ETH and UCY datasets. 

Sadeghian et al. [102] developed an interpretable GAN-based trajectory prediction model called SoPhie that combines a social attention mechanism with physical attention. For each participant, an attention module, comprising both social and physical attention mechanisms, is fed with LSTM extracted features. The output layer generates *socially* and *physically* feasible paths using a LSTM-based GAN.

**Figure 12:**
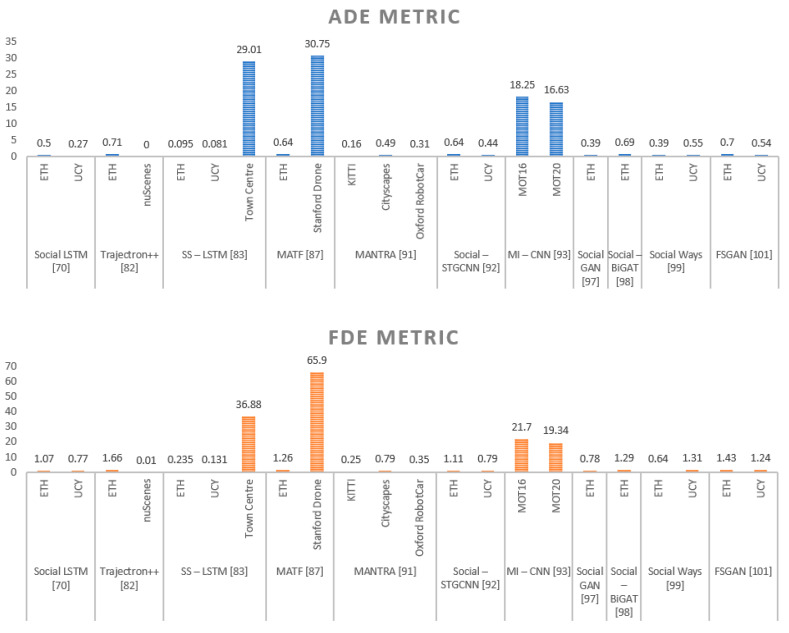
Comparative ADE and FDE metrics for each method referred in Table 2.

Refer to Table 2 for the experimental results summary of the above-mentioned deep learning paradigms for pedestrian trajectory prediction, and for a qualitative representation and comparative holistic view regarding the performance of ADE and FDE metrics for each presented method, see Figure 12.

**Table 2:** Classification of the most relative pedestrian trajectory prediction solutions, detailing prediction methods and the results.

DNN Architecture	Paper	Summary of Prediction Method	Dataset and Results (Metric)
Recurrent Neural Networks	Social LSTM [70]	The method has a novel architecture that connects the LSTMs corresponding to nearby sequences: Social pooling (S-pooling) layer to connect LSTM layer. LSTM type encoder Memory network (past memory and future memory) with RNN architecture	ETH ADE: 0.50 FDE: 1.07 NL-ADE: 0.25 UCY ADE: 0.27 FDE: 0.77 NL-ADE: 0.16
	Trajectron++ [82]	LSTM network with 32 hidden dimensions to encode a node’s ground truth future trajectory. Two nodes (History, Future) with semantic class matching. Encoder (LSTM—Edge—Map) Decoder (Gated Recurrent Unit—Gaussian Mixture Model)	ETH ADE: 0.71 FDE: 1.66 KDE NLL: 1.31 nuScenes FDE: 0.01 (1 s) KDE NLL: −5.58 (1 s)
	LSTM—Bayesian [59]	Bounding box and odometry sequences are the inputs to the sequence RNN model. This model has encoder–decoder layers with Bayesian modeling: Encoder (extract visual features to improve longer-term prediction). At the base, this encoder has CNN architecture with 10 convolutional layer and ReLU non-linearities. Decoder (to extract the odometry and visual summary vectors at every time-step).	Cityscapes MSE: 695 L: 3.97
	DESIRE [75]	This method uses the Sample Generation Module which is an encoder–decoder architecture. The following Ranking and Refinement Module adjusts the prediction samples at each time step to have more accurate predictions. The regression of prediction samples is refined with an interactive RNN layer feedback. CVAE is used to prediction of the short-term visual motion from a single image.	KITTI Error in meters/miss-rate with 1 m threshold: 0.27/0.04 Stanford Drone Pixel error at 1/5 resolution: 1.29
	SS—LSTM [83]	LSTM network with 128 dimensions using encoder and decoder architecture. Hidden states have non-linear ReLU activations layer.	ETH ADE: 0.095 FDE: 0.235 UCY ADE: 0.081 FDE: 0.131 Town Center ADE: 29.01 (0.8 s) FDE: 36.88 (0.8 s)
Convolutional Neural Networks	MATF [87]	The method uses encoder and decoder architecture. To capture the multimodal uncertainty of predictions the architecture use generator G and discriminator D. Encoder (dynamic scenes—LSTM layer, static scenes—CNN layer) Decoder (LSTM layer)	ETH ADE (Deterministic): 0.64 ADE (Stochastic): 0.48 FDE (Deterministic): 1.26 FDE (Stochastic): 0.90 Stanford Drone ADE (Deterministic): 30.75 ADE (Stochastic): 22.59 FDE (Deterministic): 65.90 FDE (Stochastic): 33.53
	MANTRA [91]	The model has encoder–decoder architecture with autoencoder system. - Encoder (learn to map past and future points into a meaningful representation) - Decoder (learn to reproduce the future) Memory network layer (past memory, future memory) to adjust predictions	KITTI ADE: 0.16 (1 s) FDE: 0.25 (1 s) Cityscapes ADE: 0.49 FDE: 0.79 Oxford RobotCar ADE: 0.31 (1 s) FDE: 0.35 (1 s)
	Social—STGCNN [92]	Spatio-temporal graph representing G = (V, A), when G is forwarded through the spatio-temporal graph CNN. Fallowing this, TXP-CNN layer is responsible with futures trajectories. P is the dimension of pedestrian position. N is the number of pedestrians. T is the number of time steps	ETH ADE: 0.64 FDE: 1.11 UCY ADE: 0.44 FDE: 0.79
	MI—CNN [93]	The method implemented encoder–decoder module by encoding and decoding the input information about pedestrians. The encoder–decoder module contains several blocks structured in convolution layer. - Encoder contains four parts: the pose, the 2D and 3D size information, historical trajectories, and the depth map. - Decoder has the kernel size and the stride similar with the encoder	MOT16 ADE: 18.25 FDE: 21.70 MOT20 ADE: 16.63 FDE: 19.34
Generative Adversarial Networks	DGMMPT [96]	Algorithm for data association in multi person tracking. Generator composed from: - encoder (Convolution-BatchNorm-ReLU layers), LSTM and - decoder (Convolution-BatchNorm-Dropout-ReLU layer) Discriminator—same as encoder layer	3D MOT 2015, AVG-Town Centre MOTA: 42.5, MOTP: 69.8
	Social GAN [97]	The network learns social norms in a data-driven approach Generator composed from: - LSTM type encoder - Pooling Module - LSTM type decoder Discriminator—same as encoder layer	ETH ADE: 0.39/0.58 FDE: 0.78/1.18
	Social—BiGAT [98]	Graph-based generative adversarial network in the form of graph attention network (GAT) that learns reliable feature representations that encode the social interactions between humans in the scene.	ETH ADE: 0.69 FDE: 1.29
	Social Ways [99]	Info-GAN plus hand-designed interaction features inspired from the neuroscience and biomechanics.	ETH: ADE: 0.39 FDE: 0.64 UCY ADE: 0.55 FDE: 1.31
	FSGAN [101]	Attentive GAN using two attention modules: physical attention and social attention.	ETH: ADE: 0.70 FDE: 1.43 UCY ADE: 0.54 FDE: 1.24

## 4. Datasets

Large datasets are indispensable resources for training and testing DNN models. To achieve this, they are annotated with ground truth information. In one possible scenario, the pedestrian is instructed to perform predetermined actions. More realistic scenarios are also used. Unfortunately, the number of data collected is limited. One can identify two major challenges in the available datasets. The pedestrian is instructed to perform predetermined actions, (stopping, using the crosswalk, crossing, etc.). Being predetermined, it does not comprise all information (the recorded pedestrian is an “actor”, the variability of his actions is nonexistent). In real life, pedestrian actions are variable, driven by events that may or may not happen (bus arrival, the crossing traffic light turning red, etc.). Real-life scenarios were used for low-level models such as detection and tracking. Unfortunately, these scenarios do not offer data needed for higher level models (for example social interactions). 

To test the pedestrian trajectory prediction systems, researchers typically use multiple datasets. They provide pedestrian images from different scenarios (boardwalks, zebra crossings, sidewalks, etc.). In these images, people are moving in different directions. Most of the detectors use color information [103], although vision-based approaches cannot collect and deliver the same level of information at night–day or low-light conditions. 

In order to compare prediction performance, there are several metrics used: final displacement error (FDE) and average displacement error (ADE) are applied on standardized prediction assignments. Average displacement error (ADE) can be defined as the average of root mean squared error (RMSE), which is calculated between the predicted trajectory location and the ground truth. This is computed every timeframe during a period of 5 s. It is known that a method that provides small ADE values has a small drift from ground truth. A method such as this can be convenient. The RMSE between the trajectory prediction and the ground truth at the last timeframe represents the final displacement error (FDE). Better long-term predictions are suggested by low FDE methods.

Although there were different datasets used in the same manner, the performance comparisons occasionally generate disputes, remaining hard to emphasize the importance of a good performance on a specific dataset or sequence, as regards a prediction algorithm. The recording of the raw data can be achieved using one or more different types of sensors: monocular cameras, stereo-cameras, radar, RGB-D cameras, LiDAR or a mix between these sensors. 

### 4.1. Traffic Capture

The Caltech Pedestrian Dataset [104] is one of the most used datasets. It contains RGB images and bounding boxes for pedestrians. Occlusion bounding boxes highlighting the visible portions of the pedestrians are also available. The Caltech Pedestrian Dataset consists of 10 h of 640 × 480, 30 FPS video taken from an urban environment driving vehicle. About 250,000 frames were annotated. They contain approximately 137 min long segments and a total of 350,000 bounding boxes use to mark about 2300 pedestrians.

The KITTI dataset [56] was also recorded from inside of a vehicle. It contains images of urban roads annotated with bounding boxes. KITTI contains depth and semantic segmentation maps and LiDAR point clouds. They enable comparisons of results obtained by different approaches using the same input. Including more than one hundred ranked methods, the KITTI pedestrian detection benchmark promotes a significant evolution in the autonomous driving vehicle field. It provides an infrastructure that allows testing and comparisons of different approaches for pedestrian detection and tracking. 

The Daimler dataset [105] provides pedestrian detection ground truth. It contains information regarding pedestrian intention. The Daimler dataset considers four different pedestrian motion types: starting to walk, stopping, crossing, and bending in. Each video sequence contains the above-mentioned labels regarding pedestrian intentions. 

The KAIST [106] employs thermal camera images (most of the datasets use RGB images only). The dataset combines them with data collected using regular cameras. The aim is to improve deep neural networks training. 

The PIE dataset [107] consists of over 6 h of driving footage captured with calibrated monocular dashboard camera Waylens Horizon equipped with a 157° wide angle lens. PIE contains HD format (1920 × 1080 px) videos delivering 30 fps. Over 300 K labeled video frames revealing 1842 pedestrian samples make PIE the largest, publicly available set suited for pedestrian behavior in traffic. 

The inD dataset [108], which is achieved using a static drone, includes over 11 K trajectories of road users, typically motorized agents. The scenarios are based on urban mobility, including scenes of road intersections or roundabouts. A similar motivation regarding the observation of the spaces between cars and other road users was performed by Ko-PER [109]. Using videos and laser scans, the trajectories of vehicles and pedestrians at a certain road intersection could be provided.

Some datasets offer data collected for training/benchmarking algorithms for autonomous vehicles (AV). They may be more difficult because of mobile data acquisition and because the trajectories are often shorter. The LCAS [110] dataset contains 28,002 Velodyne [111] scan frames acquired in one of the main buildings (Minerva Building) of the University of Lincoln, United Kingdom. Total length of the recorded data is about 49 min. Data were grouped into two classes according to whether the data were acquired from a LiDAR sensor on a mobile robot. 

The ApolloScape dataset [112] provides a larger and richer labeling. This includes stereo, per-pixel semantic labeling, holistic semantic dense point cloud for each site, lane mark labeling, instance segmentation, 3D car instance, etc. It also provides high accurate location for every frame for driving videos from multiple sites and cities. The dataset includes 1000 km trajectories for urban traffic in varying conditions and traffic densities, about 100 K image frames and 80 k LiDAR point cloud. 

The Cityscapes dataset [81] focused to 2D segmentation. The dataset contains 30 semantic classes. Detailed (5000 images) and coarse (20 k images) annotations were performed. Only one image out of each video was manually labeled. Thus, the results of video segmentation tasks (and other similar) are believed to be modest (if they can be performed). 

The BDD100 K dataset [113] contains about 100 K video sequences (raw). It represents more than 1000 driving hours and contains more than 100 million images. Similar to Cityscapes, only one image per clip is selected for annotation. About 100 K images are annotated in the bounding box level while 10 K images are annotated on the pixel level.

Another large dataset, namely The Argoverse dataset [114], was collected in the cities of Miami and Pittsburgh, in the United States of America. The recordings were made in various weather during different moments throughout the day. They contain surround view images from seven cameras and a stereo vision system. Two 32-beam Velodyne LiDAR sensors stacked vertically were used to provide 64-beam LiDAR point clouds. Synchronized frontal-view images with corresponding point clouds from the original Argoverse dataset were extracted. A timestamp tolerance of 51 milliseconds between LiDAR sweeps and images was allowed. The resulting dataset provides sets for training (about 13 k images), validation (over 5 k images), and testing (more than 4 k testing images). 

The nuScenes dataset [58] contains training data (more than 28 k) and validation (over 6 k images). The validation set is considered for testing, while the training set is split into 11 k training and 3 k validation images. The scenes were collected in the Boston area, USA and in Singapore in various weathers during different moments of the day. The dataset provides the point cloud for each image. A 32-beam roof LiDAR was used for this purpose. The dataset also provides 360° images recorded from five cameras. 

The Lyft Level 5 dataset [115] contains over 18 k frontal view images. About 12.6 k images were used for training purpose. Some 3 k images formed the validation subset while 3 k of them were selected for testing. The data was recorded around Palo Alto, USA during daytime in clear weather. This dataset provides the point cloud for each image, and 40 (or 64)-beam roof LiDAR and two 40-beam bumper LiDAR sensors were used. Five camera images covering 360° are also available in the dataset. 

The Waymo dataset [116] contains 122 k training, about 30 k validations, and around 40 k test images. It is split into 12 k, 3 k and 3 k for training, validation, and testing, respectively. The dataset contains images recorded in Phoenix, Mountain View, and San Francisco in different day moments and various weathers. The Waymo dataset also contains the combined point cloud for each image. Five LiDAR sensors (one on the roof) and several cameras were used to record this data. 

The H3D dataset [117] includes over 27 k images representing 160 crowd scenes with a total of 1.1 M 3D boxes annotated. A 360° view is used to annotate objects (for comparison, in the KITTI dataset, only front view objects are marked). 

The TRAF dataset [60] considers the categories of car, bus, truck, rickshaw, pedestrian, scooter, motorcycle, and animals. The dataset contains 13 motorized vehicles, 5 pedestrians and 2 bicycles per frame, respectively. Annotations were performed following a strict protocol, and each annotated video file consists of spatial coordinates in pixels, an agent ID, and an agent type. Camera viewpoint (front-facing/top-view) is considered to categorize dataset images. Different day moments (day, evening, and night) and different difficulty levels are considered.

### 4.2. Surveillance Capture

ETH [71] and UCY [72] are the most commonly used datasets in this area, which are based on surveillance videos of pedestrians who are walking on the footpath annotated with their location coordinates. The UCY dataset is able to deliver gaze directions, which are used to capture the pedestrian’s view angle. These two datasets include five scenes, three that are from UCY (called Univ, Zara1 and Zara2) and two from ETH (called ETH and Hotel). Overall, they involve over 1600 pedestrian trajectories, with the pedestrian’s locations being annotated every 0.4 s. In order to achieve training and testing, it uses the leave-one out cross-validation method, which assumes that the model must be trained on four different scenes, but it must be tested on the fifth. This process has to be repeated once for each scene, meaning five times. From now onward, these two datasets will be mentioned as the ETH-UCY dataset, considering the fact that they are used jointly.

Another worth-mentioning dataset is Stanford Aerial Pedestrian (SAP), sometimes known as Stanford Drone (SD) [76]. The images in this dataset provide a top-down view of road users (the images are recorded by drone). Dataset annotations include object class labels and bounding boxes. At every 0.4 s, the pedestrian’s location is annotated per one frame. The authors divided the data into training and testing, and as regards the test set, it is available for only the observed location. It can deliver the trajectories of ∼19 k walking agents in the area of a university campus, with interactions between pedestrians, cyclists, skateboarders, cars, and buses. 

The VIRAT Video Dataset [118] is a surveillance video dataset (large scale). It is designed to assess the event recognition algorithms performance using realistic scene(s). It includes aerial vehicle and stationary ground camera data. After this release, in 2018, ActEV/VIRAT [119] appeared, which is a better version of VIRAT, containing more annotations and videos. It involves over 12 h of recordings, including 12 scenes with 455 videos at 30 fps. Most of the videos are characterized by a high resolution of 1920 × 1080.

The ATC [120] dataset is based on annotations provided by 49 3D sensors for 92 days, as regards the pedestrian trajectories in the area of a shopping mall. The Town-Centre dataset [84] was developed with the purpose of visual tracking, using annotations of video footage to observe a crowded town center. It refers to ~2000 moving pedestrians walking along the street, defined by natural behaviors. Particularly, the PETS’2009 dataset [121] contains 11 sequences that are captured by eight monocular cameras, including data provided by acting pedestrians, and having various levels of density.

Table 3 concludes the presentation, containing references to 25 PTP datasets.

## 5. Discussion and Conclusions

Pedestrians need the highest protection on the road, being the most susceptible road users. The urgency of creating pedestrian protection systems is highlighted by the multitude of injuries and deaths. Pedestrian protection systems lead to multiple research problems, such as creating different types of sensors, extracting proper features from the processed sensor information, analyzing and classifying these features for the detection, and tracking of pedestrians and their behavior, which analyzes not only the pedestrians, but also the drivers, interfaces and human factors.

In this paper, we have reviewed current state-of-the-art sensors and deep learning methods applied to the pedestrian trajectory prediction problem. 

The topic is of high interest, as the number of published research papers proves. This also includes several surveys on PTP published in recent years, e.g., [122,123]. For example, an in-depth overview of human motion trajectory prediction is presented in [124]. The presentation is made from multiple perspectives, e.g., in the context of service robots or surveillance systems, whereas our work is primarily focused on PTP-based automotive applications. The works of [125,126] refer to issues, surveys, and challenges in pedestrian protection systems, highlighting the importance of infrastructure (including V2X and vehicle-to-pedestrian communication systems) and passive and active safety system designs. It partially overlaps our work, mainly in the presentation of the sensorial part, but it lacks the presentation of the DL-based algorithms associated with each particular type of sensor that we provided in Section 2. Some surveys consider the general framework of motion prediction for pedestrians and vehicles in the context the autonomous driving [127]. Although the authors have provided a brief overview of learning-based models and further propose a taxonomy categorization of DL-oriented methods, they do not refer to datasets, metrics, and experimental results as we have provided in Table 2 and Figure 12. Lastly, there are few surveys specifically oriented toward vision-based prediction using deep learning techniques. The most comprehensive is probably represented by the work of Rasouli [128], where trajectory prediction is presented among other algorithms (video, action, body motion predictions). In comparison, our presentation proposes a specific DL-oriented (CNN, RNN, and GAN) taxonomy. Therefore, the objective of this article is to provide a systematic review of the PTP DL methods in the context of available sensors applicable to the autonomous driving domain. 

Although the process of developing reliable solutions was laborious until now, a greater effort is still necessary to achieve a system that will ensure pedestrian security on streets. The selected papers were examined regarding several common factors to ensure a simple comparison for those interested in this topic.

The most popular sensor technologies used for PTP problems are radar, LiDAR, and video cameras, including a fusion between these sensors. As regards the type of information acquired, each sensor is characterized by different strengths and weaknesses. To achieve the perception of the real world, these sensors are mounted inside vehicles for traffic capture or at different street locations for surveillance purposes. They provide valued information about the motion and the position of pedestrians.

By leveraging deep learning approaches, the current systems are able to better solve the PTP problem. These methods assume a series of locations for pedestrians during the past several seconds and output a series of future locations. In our comprehensive review, we identified three main DNN architectures (RNN, CNN and GAN) best suited for the problem of pedestrian trajectory prediction. These approaches are not exclusive and often are used in hybrid combination. 

The study performed over the available PTP datasets refers to 25 heterogeny examples, containing controlled and uncontrolled scenarios, using fixed or mobile sensors. For each of them, key features (sensor type, data size, number of pedestrians, etc.) were extracted and summarize (Table 3).

Although current PTP methods have been considerably improved, they can still be upgraded for better real-world applications. This survey enables the reader to identify current challenges and future tasks or research opportunities in the domain of DL-based PTP. One of the most problematic issues is related to the ability to compare the experimental results due to the different metrics used in reporting the results: ADE, FDE minimum average or final displacement error (mADE, mFDE), dynamic time warping (DTW), modified Hausdorff distance (MHD) or negative log-likelihood (NLL). According to [124], “probabilistic metrics are preferable as they better reflect the stochastic nature of human motion”. In relation to the metrics problem, the development of new datasets should represent a constant preoccupation. They should provide an increased level of density and a higher interaction between agents. These features will lead to more accurate estimations regarding the quality of the prediction algorithms already proposed. Future methods should consider weather conditions, time of day, interactions, scene understanding, and map awareness. Prediction techniques that consider trajectories as separate processes and are not based on specific modeling of interactions may be effective on some particular datasets where the trajectories are characterized by low collision energy. Eventually, the research on sensor fusion may lead to new advancements in the field. Although today, a variety of sensors are employed for PTP implementation, a less expensive day/night video camera-based solution is preferred. 

In conclusion, DL-based PTP clearly outperforms traditional/statistical motion models and represents today a fast-growing field that opens the door to future progress in the domain by stimulating the research on the most promising solutions.

## Figures and Tables

**Table 1:** Summary of the performance aspect at each automotive sensor (radar, lidar and camera) by highlighting their pros and cons in different tasks. An adapted version based on [66].

Performance Aspect	Radar	Lidar	Camera	Fusion (Radar + Lidar + Camera)	Metrics
Object detection	High	High	Moderate	High	Accuracy
Object classification	Low	Moderate	High	High	Accuracy
Distance estimation	High	High	Moderate	High	Accuracy
Edge detection	Low	High	High	High	Sensitivity
Lane tracking	Low	Low	High	High	Linearity
Visibility range	High	Moderate	Moderate	High	Resolution
Bad weather performance	High	Moderate	Low	High	Accuracy
Low illumination	High	High	Moderate	High	Sensitivity

**Table 3:** Overview on available datasets divided by type, publishing year, location, etc.

Dataset	Type	Year	Sensor	Size	3D Boxes	No. of Pedestrians	Annotated Frames	Locations	View
UCY [72]	Surveillance	2007	RGB Camera	29.5 min	No	1456	No	Nicosia	Bird view
The Town-Centre dataset [84]	Surveillance	2009	CCTV Camera	-	No	2200	Yes	Oxford	Bird view
PETS’2009 dataset [121]	Surveillance	2009	CCTV Camera	-	No	4307	Yes	Reading	Bird view
The Caltech Pedestrian Dataset [104]	Traffic	2009	RGB Camera	10 h	No	2300	Yes	Los Angeles	Terrestrial
ETH [71]	Surveillance	2009	RGB Camera	25 min	No	750	Yes	Zurich	Terrestrial
VIRAT Video Dataset [118]	Surveillance, Activities	2011	Stereo Camera	29 h	No	-	Yes	USA	Bird view
KITTI [56]	Traffic	2012	RGB Camera, LIDAR, GPS/IMU	1.5 h	Yes	30	Yes	Karlsruhe	Terrestrial
ATC [120]	Surveillance	2013	RGB Camera, LiDAR	24 h	No	407	No	Osaka	Bird view
Daimler dataset [105]	Traffic	2013	RGB Camera	-	No	68	Yes	Ulm	Terrestrial
Ko-PER [109]	Traffic	2014	RGB Camera, LiDAR	6.2 min	Yes	38	Yes	Ulm	Bird view
KAIST [106]	Traffic	2015	RGB Camera, Infrared Camera	-	No	1182	Yes	Seoul	Terrestrial
Stanford Drone (SD) [76]	Surveillance	2016	RGB Camera	-	No	3297	Yes	California	Bird view (drone)
Cityscapes [81]	Traffic	2016	Stereo Camera, RGB Camera, GPS/IMU	-	Yes	-	Yes	Germany	Terrestrial
L-CAS [110]	Traffic	2017	RGB Camera, LiDAR	49 min	Yes	6140	Yes	Lincoln	Terrestrial
BDD100K dataset [113]	Traffic	2017	RGB Camera, GPS/IMU	1000 h	No	-	Yes	New York, San Francisco	Terrestrial
ActEV/VIRAT [119]	Surveillance	2018	Stereo Camera	12 h	No	-	Yes	USA	Bird view
ApolloScape dataset [112]	Traffic	2018	RGB Camera, LiDAR, GPS/IMU	2 h	Yes	-	Yes	China	Terrestrial
inD dataset [108]	Traffic	2019	Camera	10 h	No	~4000	Yes	Aachen	Bird view/drone
PIE dataset [107]	Traffic	2019	RGB Camera	6 h	No	1842	Yes	Toronto	Terrestrial
Argoverse [114]	Traffic	2019	RGB Camera, LiDAR, GPS/IMU	320 h	Yes	10,726	Yes	Miami, Pittsburgh	Terrestrial
nuScenes [58]	Traffic	2019	RGB Camera, LiDAR, GPS/IMU, Radar	6 h	Yes	719	Yes	Boston	Terrestrial
Lyft Level 5 [115]	Traffic	2019	RGB Camera, LiDAR, GPS/IMU, Radar	1118 h	Yes	-	Yes	Palo Alto	Terrestrial
Waymo [116]	Traffic	2019	RGB Camera, LiDAR, GPS/IMU, Radar	10 h	Yes	-	Yes	USA	Terrestrial
H3D [117]	Traffic	2019	RGB Camera, LiDAR, GPS/IMU, Radar	0.77 h	Yes	-	Yes	San Francisco	Terrestrial
TRAF [60]	Traffic	2019	RGB Camera	-	No	5	Yes	-	Terrestrial

## Data Availability

Not applicable.
